# The effects of weather on the spread of COVID-19: evidence from Ghana

**DOI:** 10.1186/s42269-021-00484-3

**Published:** 2021-01-12

**Authors:** Eric N. Aidoo, Atinuke O. Adebanji, Gaston E. Awashie, Simon K. Appiah

**Affiliations:** 1 Department of Statistics and Actuarial Science, Kwame Nkrumah University of Science and Technology, Kumasi, Ghana; 2 KNUST-Laboratory for Interdisciplinary Statistical Analysis (KNUST-LISA), Kumasi, Ghana; 3 Department of Mathematics, Kwame Nkrumah University of Science and Technology, Kumasi, Ghana

**Keywords:** COVID-19, Weather, Generalized additive model, Linear and nonlinear relationships

## Abstract

**Background:**

Climatic factors have been shown to influence communicable disease dynamics especially in tropical regions where temperature could swing from extreme heat and dryness to wet and cold within a short period of time. This is more pronounced in the spread of airborne diseases. In this study, the effect of some local weather variables (average temperature, average relative humidity, average wind speed and average atmospheric pressure) on the risk of Severe Respiratory Syndrome Coronavirus 2 (SARS-CoV-2) in Ghana is investigated. The daily confirmed new COVID-19 cases were compiled from the Ghana Health Service and the weather data extracted from Weatherbase. The type of relationship between the climatic variable and risk of spread were explored using the Generalized Additive Model (GAM).

**Results:**

Results obtained showed that wind speed and atmospheric pressure have positive linear relationship with the spread of infection an increase in the risk of COVID-19 spread. In addition, the risk of spread fluctuates for temperature between 24 and 29 °C but sharply decreases when average temperature exceeds 29 °C. The risk of spread of COVID-19 significantly decrease for relative humidity between 72 and 76% and leveled afterwards.

**Conclusion:**

The results indicate that wind speed and pressure have a positive linear relationship with the risk of spread of COVID-19 whilst temperature and humidity have a non-linear relationship with the spread of COVID-19. These findings highlight the need for policy makers to design effective countermeasures for controlling the spread as we are still within the low temperature season.

## Background

Coronavirus disease 2019 (COVID-19) first identified in Wuhan, China in December 2019 (Liu et al. 2020) has become a global public health concern having been declared a global pandemic by World Health Organization (WHO) on March 11, 2020 (WHO 2020). Infection of COVID-19 could present with a wide spectrum of symptoms inform of fever, cough, sore throat, diarrhea, fatigue, difficulty in breathing, kidney failure and possible fatality (WHO 2020; Linton et al. 2020). As of August 5, 2020, data from WHO have shown over 18,354,342 confirmed cases and 696,147 deaths have been reported globally (WHO 2020). Due to rapid spread of the disease, many countries implemented different mitigation and suppression programmes. Different combinations of measures such as closure of international boarders, partial/total lockdown of a country or a city, ban on social gatherings, frequent hand washing with soap under running water, physical distancing, quarantine and isolation were introduced as immediate response to the outbreak. Testing, contact tracing and treatment, compulsory wearing of face masks in public spaces were additional measures subsequently or simultaneously introduced.

Ghana reported its first two confirmed COVID-19 cases on March 12, 2020 (GHS 2020), since then, the number of cases in Ghana has been on the increased. An initial slow exponential growth during the partial lockdown of Greater Accra and Ashanti regions has been followed by a more rapid growth in cases following the lifting of some suppression and control measures. As of August 5, 2020, data from Ghana Health Service (GHS) shows 39,075 confirmed cases and 199 deaths recorded across the 16 administrative regions of Ghana (GHS 2020).

Due to threat posed to human and socio-economic wellbeing by COVID-19, different studies have been conducted to model the spread and understand the contribution of potential drivers for the development and implementation of evidence-based public health and disease control policies. One of the potential predictors of COVID-19 infection that has received much attention in recent times is weather (climate). For instance, D’Amato, Cecchi (D’Amato et al. 2014) argue that respiratory disease such as SARS can be predicted by certain weather conditions. In a recent study Prata, Rodrigues (Prata et al. 2020), the relationship between weather and COVID-19 cases in Brazil was investigated using GAM. The study established a negative relationship between temperature and the spread of COVID-19. A study by Ahmadi, Sharifi (Ahmadi et al. 2020) also established that humidity and wind speed have negative relationship with the infection rate of COVID-19 in Iran.

A significant relationship between weather and the spread of COVID-19 has been established in existing literature. However, most of such studies have been conducted in America (Bashir et al. 2020; Runkle et al. 2020), Europe (Menebo 2020; Briz-Redón and Serrano-Aroca 2020), Asia (Liu et al. 2020; Ahmadi et al. 2020) and Australia (Ward et al. 2020) and limited information exist on the African continent which has been predicted as the next epicenter for COVID-19. The rapid spread of the disease poses a serious burden due to its weak healthcare systems characterizing most African countries, especially the sub-Saharan African countries. Although, knowledge from studies conducted in other continents could be used as a basis for policy formulation in general, country-specific studies may be required due to variations observed in the spread of COVID-19 as well as the weather characteristics among different countries. This study seeks to contribute to the knowledge on the effect of weather on the spread of COVID-19 infection in Ghana. The findings from could be helpful in assisting public health professionals to develop control measures.

## Methods

### Study area and data

The study covers all the 16 major administrative regions of Ghana. Ghana is in the sub-region of West Africa located between latitude 7.9465° N and 1.0232° W. The country has total land area of 239,567 km^2^ with an estimated population of 30,280,482 (GSS 2020). The territorial border of Ghana from the southern part is bounded by the Gulf of Guinea and Atlantic Ocean. The climatic condition of Ghana is tropical with average daily temperature ranging from 30 °C during the day to 24 °C at night (UNDP 2020).

The data used in this study consist of daily new confirmed cases of COVID-19, daily average temperature (°C), daily average relative humidity (%), daily average wind speed (km/h) and daily average atmospheric pressure (hpa). Since Greater Accra region is the epicenter of COVID-19 in Ghana, the weather data of Greater Accra was used as proxy for the averages of weather variables in Ghana. The data spanned the period from March 12 to July 31, 2020. The starting date of the data corresponds to the day the first COVID-19 confirmed cases were identified. The daily new cases of COVID-19 data were extracted from GHS (GHS 2020) and the weather data were extracted from Weatherbase (2020). For each variable there were 115 observations.

### Model specification

In this study, the effect of weather on daily new cases of COVID-19 was determined using GAM, semi-parametric extension of generalized linear model (GLM) to account for a nonlinear relationship between the dependent variable and a set of covariates. The choice of the model is influenced by the nonlinear relationship that is believed to exist between weather and infectious diseases (Prata et al. 2020; Zhu and Xie 2020; Colón-González et al. 2013).

Let $y_{i}$ represents the new cases of COVID-19 for day *i* and $E(y_{i} ) = \mu_{i}$ represents the expected number of cases, then the GAM is defined by Wood (2017) as:

1 $$g(\mu_{i} ) = X_{i}^{*} \theta + f_{1} (x_{i1} ) + f_{2} (x_{i2} ) + \cdots + f_{m} (x_{i\,m} )$$

where $g(.)$ is a log link function of the expectation $\mu_{i}$, $X_{i}^{*}$ is a row of the model matrix for any strictly parametric model component, $\theta$ represent the corresponding parameter vector, $x_{ij} (j = 1,2, \ldots ,m)$ is the covariates (weather variables), and $f_{j}$ are smoothing functions. The estimate of the smoothing functions $f$ can be represented in a regression spline form with known *basis* functions. Let $b_{k} (x)$ represents the *k*^th^ such *basis* function, then $f$ can be expressed as a linear model defined as:

2 $$f(x) = \sum\limits_{k = 1}^{q} {b_{k} (x)\beta_{k} }$$

where $\beta_{k}$ represents an unknown smoothing parameter, which is to be estimated. The structure of the smooth function was estimated based on basis function from the Gaussian process model (Kammann and Wand 2003). This type of basis function was selected in order to account for potential temporal autocorrelation in the residuals of the model. The smoothing parameters were estimated using restricted maximum likelihood. Within the GAM framework several families of distributions may be assumed for the error structure depending on the nature of the response variable. In this study, a Poisson distribution was assumed for the error structure. The inclusion of the variables and the selection of the final model were based on Akaike Information Criterion (AIC) and Bayesian information criterion (BIC). All the analysis ware conducted using *R* version 4.0.2 (Team 2020).

## Results

### Descriptive analysis

**Fig. 1:**
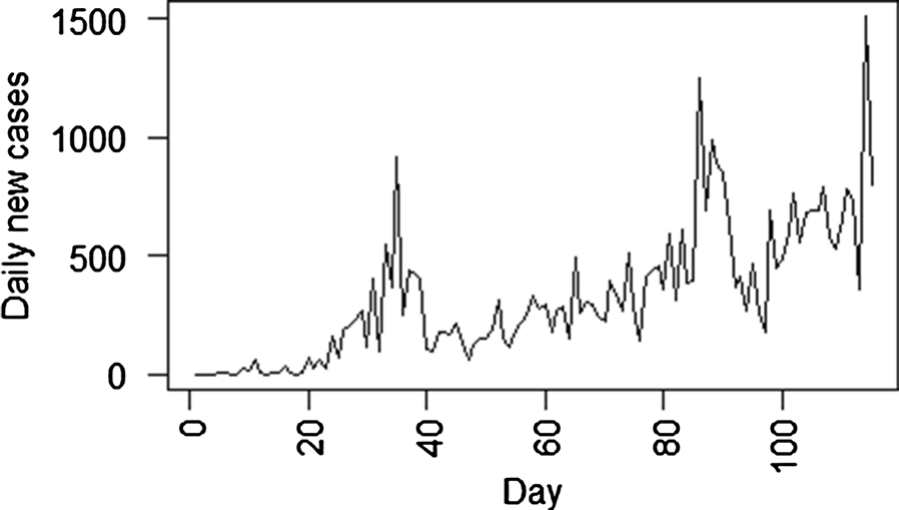
Daily new cases of COVID-19 reported during the study period

The plot of new COVID-19 cases reported in Ghana for the study period depicts an increasing trend (Fig. 1). The highest peak of new cases of 1513 was observed on July 30, 2020. The characteristics of the COVID-19 new cases and the weather variables used in this study are summarised in Table 1. An average of 329 new cases of COVID-19 with a standard deviation of 286 was observed during the study period (Table 1). The summary also shows that the distribution of the observed new cases of COVID-19 is positively skewed.

**Table 1:** Descriptive statistics of daily new cases of COVID-19 and the weather variables

Variable	Minimum	LQ	Median	Mean ± SD	UQ	Maximum
Daily new cases	1.00	117.00	271.00	328.80 ± 286	465.00	1513.00
Average Temperature (°C)	24.90	26.10	26.90	27.21 ± 1.38	28.60	29.70
Humidity (%)	72.50	77.60	81.00	80.15 ± 2.86	82.50	85.20
Wind speed (km/h)	10.10	13.00	14.50	14.63 ± 2.14	16.10	19.80
Pressure (hpa)	1010.00	1011.00	1012.00	1013.00 ± 1.68	1014.00	1015.00

SD represents standard deviation; LQ and UQ represent the lower and upper quartile respectively

**Fig. 2:**
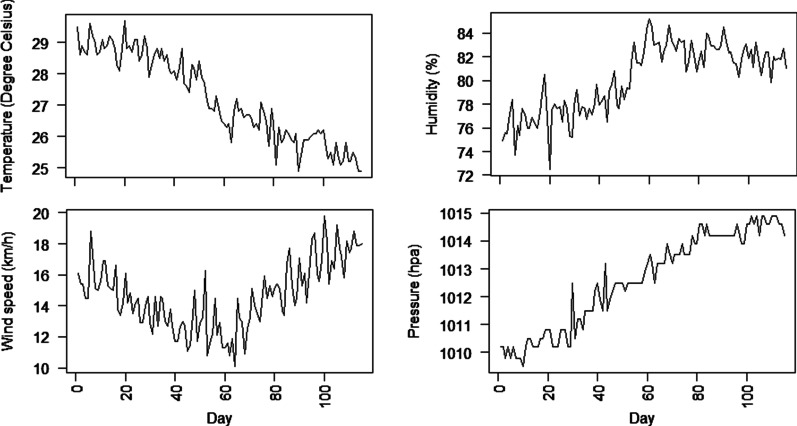
Daily average temperature, humidity, wind speed and atmospheric pressure observed during the study period

The daily average temperature observed during the study period ranged between 24.9 and 29.7 °C. In addition, the average humidity observed varied between 72.5 and 85.2% whilst average wind speed varied between 10.1 km/h and 19.8 km/h. On average the pressure observed for any given day was 1013hpa. The average temperature showed a decreasing pattern whilst humidity showed an increasing pattern during the study period (Fig. 2). During the study period, the wind speed decreased from March to June and increased afterwards whilst the average atmospheric pressure showed an increasing pattern over the period.

### Generalized additive model specification

**Fig. 3:**
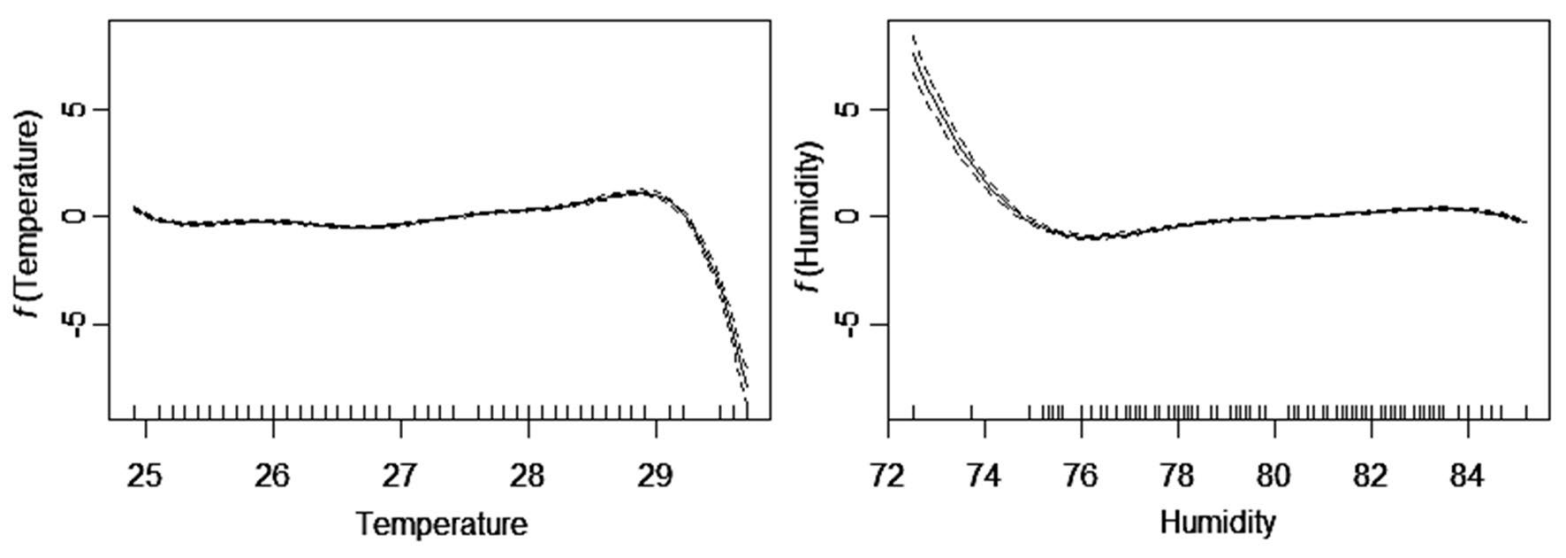
Estimated smooth curves (solid line) from GAM for average temperature (left panel) and average humidity (right panel). The dashed lines denote 95% confidence level

The effect of weather on COVID-19 infections was explored using generalized additive model with the error structure assumed to be Poisson distribution. The estimated parameters of the parametric and smoothing function terms are presented in Table 2. The estimated non-linear curves derived from the model are shown in Fig. 3. From the estimated model, average wind speed and average atmospheric pressure were found to be linearly related to the daily new cases of COVID-19 whilst the average temperature and average relative humidity have a non-linear relationship with daily new cases of COVID-19. All the variables were found to be significantly related to the daily new cases of COVID-19 at 5% level.

**Table 2:** Parameters’ estimate of the fitted GAM

Variable	Estimates	*P*-value
*Parametric coefficients*		
Intercept	− 45.81	< 0.001
Wind speed	0.010	0.010
Pressure	0.458	< 0.001

Variable	EDF^a^	*P*-value
*Smooth terms*		
*f* (average temperature)	6.984	< 0.001
*f *(humidity)	5.963	< 0.001

^a^EDF represents effective degree of freedom

## Discussion

The developed model suggests that there exists a positive linear relationship between wind speed and the spread of COVID-19. That is, the risk of spread of COVID-19 significantly increase for 1 km/h increase in wind speed. This result might be because high wind speed is likely to circulate any suspended respiratory droplet in the air thereby increasing the possibility of inhalation by people who are exposed to such environment. This result supports the existing findings (Şahin 2020) that the virus spread increase when there is high wind speed. There are however some studies which reported no significant relationship (Menebo 2020) and negative relationship (Ahmadi et al. 2020; Rosario et al. 2020) between wind speed and the spread of COVID-19. This difference seems to reflect the nature of the local environment and the context where those studies were conducted.

The results also revealed that atmospheric pressure is positively related to the spread of COVID-19. That is, the risk of spread of COVID-19 is more likely to increase for a 1 hpa increase in atmospheric pressure (Table 1) and the result confirms the findings of existing study in China (Zhu et al. 2020). The positive relationship between atmospheric pressure and the spread of COVID-19 may be explained by the fact that areas with high pressure are moistier compared to low pressure areas thereby making pathogens more active and invasive to humans.

With respect to average temperature, the results confirm that there exists a non-linear relationship between temperature and the spread of COVID-19 (Fig. 2). That is, the spread of COVID-19 is at more risk when the temperature fluctuates between 24 and 29 °C. However, the risk sharply decreases when average temperature exceeds 29 °C. This results is in agreement with a number of studies (Liu et al. 2020; Wang et al. 2020) that concluded that warm temperature tends to decrease infection rate of COVID-19. The direction of the results may be influenced by the fact that the survival pattern of the virus and its transmission routes such as respiratory droplets may be influenced by temperature. That is, high temperature is likely to reduce the activeness of the virus and their biological interaction with humans as observed in other respiratory coronavirus studies (Casanova et al. 2010; Cai et al. 2007; Gardner et al. 2019). Another study (Menebo 2020) also found contradicting results on the effect of temperature on the spread of COVID-19. Possible explanation according to Menebo (2020) is due to the fact that people are likely to stay at home during cold weather than when the sun is shining thereby reducing person-to-person contact which is one of the major route of spreading the virus.

With respect to humidity, the results show that it has a non-linear relationship with the spread of COVID-19. The risk of spread of COVID-19 significantly decreases for humidity between 72 and 76% and remain leveled afterwards. This result may be influenced by the fact that the survival pattern of the virus is motivated in contaminated areas with low relative humidity (Ahmadi et al. 2020). The results also confirms a study by Liu et al. (2020) who argued that low humidity favours the risk of transmission of COVID-19.

## Conclusions

This study provides important information regarding the effect of weather on the spread of COVID-19 in Ghana. The results of this study show that weather components such as temperature, relative humidity, wind speed and atmospheric pressure significantly influences the spread of COVID-19 infections in the Country. The results indicate that wind speed and pressure have a positive linear relationship with the risk of spread of COVID-19 whilst temperature and humidity have a non-linear relationship with the spread of COVID-19.

The study has some limitations, which need to be considered when interpreting the findings. For instance, the risk of spread of infections may be influenced by government interventions such as partial lockdowns of some selected cities in Ghana. Other variables such as socio-demographical characteristics were also in not considered.

## Data Availability

The dataset used and/or analysed during the current study are available in the public domain.

## Abbreviations

SARS-CoV-2: Severe respiratory syndrome coronavirus 2
COVID-19: Coronavirus disease 2019
WHO: World Health Organization
GHS: Ghana health service
GAM: Generalized additive model
GLM: Generalized linear model
AIC: Akaike information criterion
BIC: Bayesian information criterion
